# Model for Predicting the Micro-Grinding Force of K9 Glass Based on Material Removal Mechanisms

**DOI:** 10.3390/mi11110969

**Published:** 2020-10-29

**Authors:** Hisham Manea, Xiang Cheng, Siying Ling, Guangming Zheng, Yang Li, Xikun Gao

**Affiliations:** 1 School of Mechanical Engineering, Shandong University of Technology, Zibo 255000, China; hisham.manea@sdut.edu.cn (H.M.); zhengguangming@sdut.edu.cn (G.Z.); liyang0918@163.com (Y.L.); xkg2550@163.com (X.G.); 2 Mechanical Engineering Department, Faculty of Engineering, Sana’a University, Sana’a 12544, Yemen; 3 Key Laboratory for Precision & Non-traditional Machining of Ministry of Education, Dalian University of Technology, Dalian 116023, China; lingsy@dlut.edu.cn

**Keywords:** K9 glass, mathematical model, grinding force, brittle fracture, ductile–brittle transition, active grains number

## Abstract

K9 optical glass has superb material properties used for various industrial applications. However, the high hardness and low fracture toughness greatly fluctuate the cutting force generated during the grinding process, which are the main factors affecting machining accuracy and surface integrity. With a view to further understand the grinding mechanism of K9 glass and improve the machining quality, a new arithmetical force model and parameter optimization for grinding the K9 glass are introduced in this study. Originally, the grinding force components and the grinding path were analyzed according to the critical depth of plowing, rubbing, and brittle tear. Thereafter, the arithmetical model of grinding force was established based on the geometrical model of a single abrasive grain, taking into account the random distribution of grinding grains, and this fact was considered when establishing the number of active grains participating in cutting N_d-Tot_. It should be noted that the tool diameter changed with machining, therefore this change was taking into account when building the arithmetical force model during processing as well as the variable value of the maximum chip thickness a_max_ accordingly. Besides, the force analysis recommends how to control the processing parameters to achieve high surface and subsurface quality. Finally, the force model was evaluated by comparing theoretical results with experimental ones. The experimental values of surface grinding forces are in good conformity with the predicted results with changes in the grinding parameters, which proves that the mathematical model is reliable.

## 1. Introduction

Presently, optical glass materials like BK7 or K9 are broadly applied in the production area of various optical accessories for use in visual products or engineering appliances, such as mirrors, prisms, lenses, automobile, aerospace, and panes due to its superior scratch impedance and light transmission properties [1,2,3]. Therefore, exploring and developing efficient mechanical machining technology of optical glass has become a very important theme. K9 glass is a representative of hard and brittle materials (HBM) [4], which is prone to fracture and damage during machining due to its distinctive features, that is, fragility, resistance, hardness, strength, and alchemical stabilization. As a result, cracks and pits are easily formed on the workpiece surface, affecting the surface quality of the workpiece and the performance of the device [5,6,7]. Nowadays and because of all these factors, most HBM are processed by grinding due to its high efficiency, but the damage below the surface introduced during grinding has always been a bottleneck problem in machining [8,9]. The most important factor is grinding force, consequently, research on establishing and controlling the grinding force of HBM is particularly important for machining HBM to enhance the grinding efficiency and to upgrade the grinding tool performance [10,11,12,13]. The grinding force is closely linked with the quality prediction of the grinding tool surface, geometrical accuracy, and the technique of removing HBM. A lot of studies on micro-grinding force and material removal techniques of HBM have been conducted.

Liu et al. [14] mathematically constructed a force model for the carbon fiber material, assuming that the material removal will behave through a brittle regime, the force model was constructed through the maximum depth of indentation of a single grain in the material workpiece according to the brittle rip theory. A comparable model for K9 optical glass was built by Zhang et al. [15], depended on the mechanism of the indentation rip. The previous scholars have been repealed the forces in the ductile modes as well as neglecting the friction affection in the grinding force. Also, Sun et al. [16] proposed an arithmetical model of the grinding force for Zerodur glass in both brittle and ductile regions, considering the material removal mechanism and the influence of the frictional force. However, this model repealed the fact that not all grits are active during grinding, where the grits are randomly distributed on the wheel with their specific height and width, some of them are active and others have a free ride on the wheel. Moreover, Zhang et al. [17] proposed an arithmetic force model for Silica and Ceramics; the material removal mechanism was separated into two regimes, that are sliding and plowing grinding modes, then the scholars improved the tangential, normal, and radial force models. However, the arithmetic model has not acquired enough attention as the relative motion between the machine tool and the workpiece diverges. Xiao et al. [18] suggested a theoretical model represent the grinding force in the ductile–brittle modes for zirconia material, consideration of brittle–ductile transformation mode, whereas the force model canceled the friction leverage during grinding. Furthermore, Badger and Torrance [19] have been evolved two techniques for predicting the grinding force from wheel surface topography. The first method depends on Chalen and Oxley’s 2 D sliding-line domain model of the touch between grinding wheel grain and workpiece surface, while the other method depends on Xie’s and Willams 3 D model which creates a chain of channels on the workpiece.

With the concentration on the grinding of glass materials, many researchers have recently suggested mathematical models of grinding force, as well as the study of significant parameters to improve the quality and precision of grinding processes. Chen et al. [20] investigated a reasonable grinding technique for silica glass and acquired the optimal force model identical to the best subsurface fineness; however, the model did not reveal the random distribution of the grinding grains which plays an important role in the model force. Su et al. [21] predicted a model to express the grinding force of silica glass; the model studied how the normal and tangential (NT) forces are affected by variation in the grinding parameters. Then the scholars, after the experimental verification, showed that the force model can represent the grinding force; but the model has not considered the random distribution of the active grits and the changes in the maximum chip thickness accordingly. Zhang et al. [22] kinematically studied the micro-end grinding force of fused silica glass of ultrasonic-assisted through modeling and emulation of abrasive paths. The force model considered the ductile and brittle modes, but the force of the brittle region in the surface-grinding process is yet ambiguous. Consequently, previous studies lacked adequately representative the precise force model of the grinding process for HBM, several important reasons that are the instantaneous variation of grinding space and time as well as the mechanisms of material removal.

In the current article, K9 optical glass is considered a research object to represent the grinding force of HBM. Firstly, theoretical modeling of the micro-grinding force is developed for a single abrasive grit; because of this situation, the maximum chip thickness a_max_ is a dynamic concept in the grinding process, so every single active grain creates a different value of a_max_. Thereafter, the study constructs the force model in the elastic, plastic, and brittle fracture modes, where the material removal mode is permanent depending on the chip thickness value. The study considered the frictional force generated between the wear protrusion of grits and the grinding face, changes in the value of the frictional coefficient produced by the flux of workpiece over the touch grinding face, the actual active grains which participate in processing, and the reduction in the grinding tool during machining which directly affects the amount of removal chip thickness. Subsequently, the grinding experiments are carried out with a PCD grinding tool and the empirical formulas of NT grinding forces are analyzed; the experimental measurement results are compared to the model prediction values. Finally, the comparative study confirms that the force model can represent the surface grinding force of K9 optical glass.

## 2. Analysis of Surface Grinding Process

Clarifying the factors of the surface grinding process is a primary task to analyze and correctly establish the grinding force model through the different mechanisms of the material removal modes.

**Figure 1:**
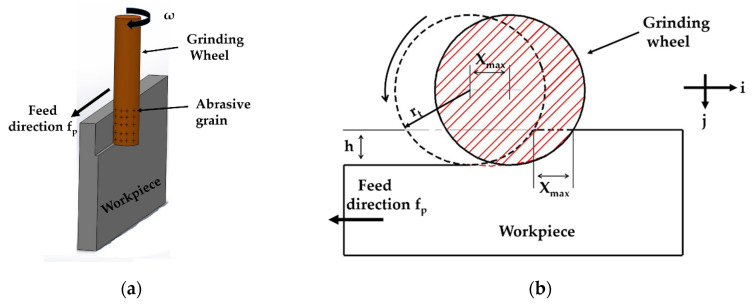
The grinding process: (**a**) Radial surface grinding mechanism; (**b**) grinding zone parameters.

Figure 1 clarifies the mechanism and the main parameters of the radial surface grinding process. The wheel rotates at an angular speed of ω and advances in the direction of the *x*-axis towards the interior surface of the workpiece at a feed rate of f_p_.

### 2.1. Displacement and Motion of Single Abrasive Grit

As displayed in Figure 1, the relative displacement of a single grit S with respect to the workpiece is identified as a function of time represented by Equation (1):

(1) $$\vec{S\left(t\right)}=\left(r_{t}\sin\left(\omega t\right)+f_{p}t\right)i+\left(r_{t}-r_{t}\cdot \cos\left(\omega t\right)\right)j$$

where r_t_ is the radius of the grinding tool, f_p_ is the feed rate, ω is the angular spindle speed, and t defined as the grinding time.

The relative velocity of a single grain to the workpiece V_r_ is calculated by the first time derivative of the displacement formula as:

(2) $$\vec{V_{r}\left(t\right)}=\frac{\partial S}{\partial t}=\left(r_{t}\cdot \omega \cdot \cos\left(\omega t\right)+f_{p}\right)i+\left(r_{t}\cdot \omega \sin\left(\omega t\right)\right)j.$$

### 2.2. Chip Thickness Model Establishment

**Figure 2:**
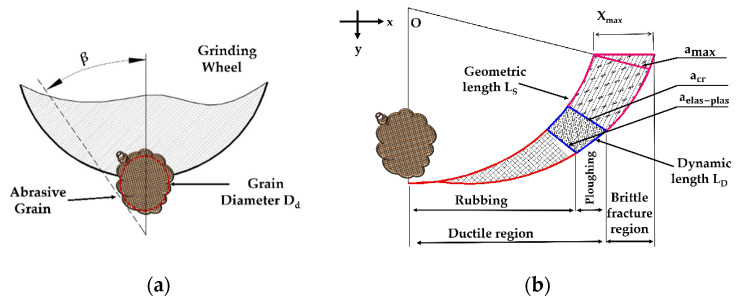
Morphology and motion parameters of abrasive grain: (**a**) Abrasive grain geometry; (**b**) mechanisms of material removal.

The most important step in establishing the force model of the surface grinding process is identifying the regions of the material removal. Therefore, Section 2.2 discusses in detail how to define the various modes of material removal. Figure 2a presents the geometry of the abrasive grain and clarifies the active grain parameters such as semi-apex angle β of the active grain and the average diameter of the active grain D_d_ which was acquired by gauging the average radius rate of abrasive extremity using AFM.

As shown in Figure 2b, the entire process of the surface grinding on the interactivity between the grits and the workpiece is classified as ductile region, ductile–brittle transition, and brittle region. The ductile region is divided into three modes, namely, chip formation, elastic, and plastic. Accordingly, the mechanisms of the material removal are classified based on the elastic-to-plastic chip thickness a_elas-plas_, critical chip thickness at ductile-to-brittle transition a_cr_, and maximum chip thickness a_max_; can be modeled as follows [23]:

(3) $$\{\begin{array}{c} \left\{\begin{array}{c} a_{c}<a_{\text{elas-plas}}\;(\mathrm{rubbing})\\ a_{\text{elas-plas}}<a_{c}<a_{\mathrm{cr}}\;(\mathrm{ploughing}) \end{array}\right\}\;\mathrm{Ductile}\;\mathrm{region}\\ a_{c}>a_{\mathrm{cr}}\;\left(\mathrm{Brittle}\;\mathrm{region}\right)\} \end{array}.$$

#### 2.2.1. Chip Thickness of the Elastic-Plastic Transition 

Correct modeling of the grinding force needs to consider the recovery and deformation of the elastic mode, in particular at the level of the initial contact zone [23]. So, the designation of the elastic zone needs to locate the transferring from elastic-to-plastic, which is pointed out based on the contact theory of Hertz’ and expressed in Equation (4) [24].

(4) $$a_{\text{elas-plas}}={\left(\frac{2\pi H_{p}}{7}\cdot \frac{\left(1-{\upsilon_{p}}^{2}\right)}{E_{p}}\right)}^{2}\cdot r_{d}$$

where r_d_ is the mean radius of the active diamond grain, H_p_ is the material hardness, υ_p_ and E_p_ are the Poisson’s ratio and Young’s modulus of the workpiece respectively.

#### 2.2.2. Chip Thickness of the Ductile–Brittle Transition 

Studying the critical depth of cut a_cr_ is the main key for controlling surface grinding of HBM, as the chip thickness must remain under the a_cr_ to stay grinding in the ductile region [25]. To easily locate the transition point from ductile-to-brittle mode, it is supposed that the grinding parameters do not affect the workpiece specifications during machining, as a result, the critical chip thickness from ductile-to-brittle fracture a_cr_ is expressed by Equation (5) which identified through material properties of the workpiece material [26,27].

(5) $$a_{\mathrm{cr}}=\varepsilon \left(\frac{E_{p}\;}{H_{p}}\right){\left(\frac{K_{\mathrm{IC}}\;}{H_{p}}\right)}^{2}$$

where ε is a unitless constant (ε = 0.15) [28,29], H_p_ is the material hardness, E_p_ is Young’s modulus, and K_IC_ tensile strength.

#### 2.2.3. Maximum Chip-Thickness 

In the existing study, the maximum chip thickness a_max_ for two uninterrupted grits is established according to the grinding parameters and expressed in Equation (6) [25,30].

(6) amax=(34)16(πCG)13(4fp·rd20.3ω·rt·tanβ)12(h2rt)14=3.031·rdCG13·(fpω·tanβ·hrt3)12

where β is defined as the half-oblique angle of a single active grit (suggested as 60° [31]), h is the grinding depth, C_G_ is the size fracture of grinding grit.

**Figure 3:**
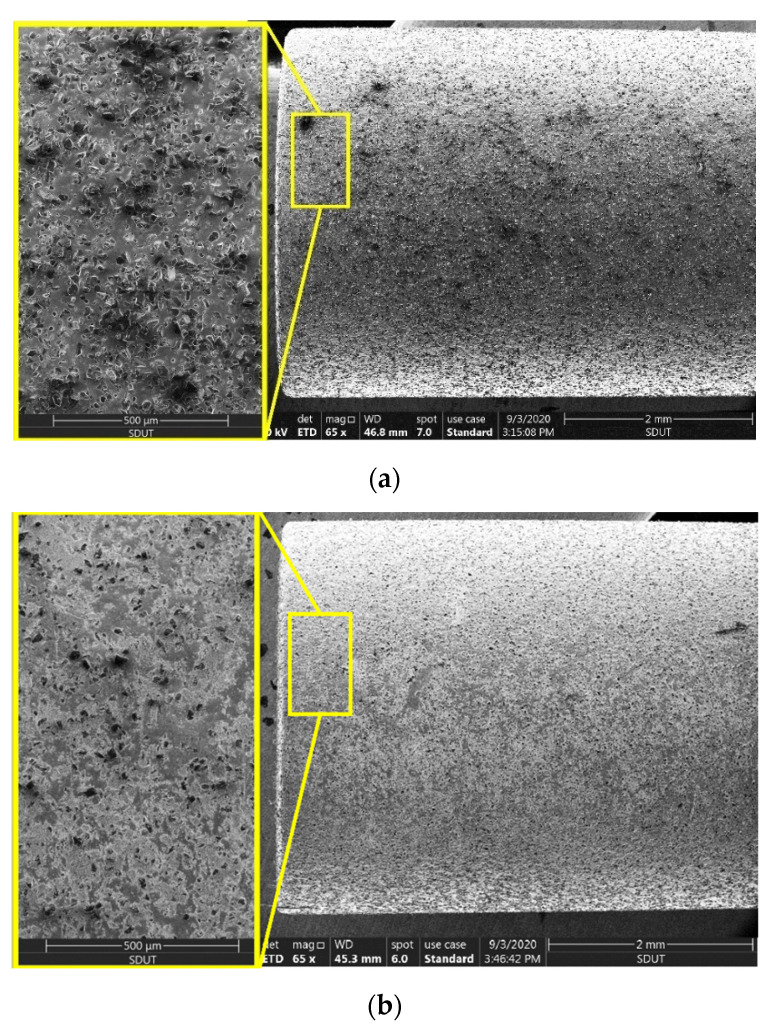
SEM captures clarify the wheel surface topography: (**a**) Unused grinding wheel; (**b**) after machining.

Figure 3a shows the topography of the grinding wheel surface which clearly shows the density of the grinding grains before the grinding process, while Figure 3b shows the effect of grinding on the grains that are dislocated or removed by machining which led to a decrease in the diameter of the grinding wheel accordingly. In general, the diameter of the grinding tool is affected by the high speed, therefore, the diameter decreases by increasing the spindle speed, feed rate, or grinding depth, so the a_max_ is a dynamic concept that can be calculated through Equation (7) [32,33].

(7) amax=3.031·rdCG13·(fpω·tanβ·hrt3)12−Xmax22rt.

The cutting path of the cutting edge of the previous grain moves from the first touchpoint to the workpiece surface. The linear distance from the first to the last touchpoints is equal to the amount of translation of the workpiece X_max_ during the interval between the turning of adjacent cutting edges as displayed in Figure 1b, X_max_ can be calculated by Equation (8).

(8) $$X_{\max}=\frac{\lambda_{r}\cdot f_{p}}{{\omega r}_{t}}$$

where λ_r_ is the average space between any two active grains calculated by Equation (9) [31].

(9) $$\lambda_{r}=\left(\sqrt{\frac{\pi }{C_{G}}}-2\right)\cdot r_{d}.$$

By substitution Equations (8) and (9) into Equation (7), the a_max_ is expressed as

(10) amax=3.031·rdCG13·(fpω·tanβ·hrt3)12−(πCG−2)2·(rd22rt)·(fpωrt)2.

### 2.3. Clarifying the Various Grinding Areas

Identification of the NT grinding areas of a single grain, whether projected or contacting, is a very important task to accurately establish the NT grinding forces of a single grit. The touch depth is almost equivalent to the semi-value of the depth of cut when the grit begins to touch the working surface in the elastic region [24]. Meanwhile, the touch depth is almost equivalent to the depth of cut during plastic mode [34].

**Figure 4:**
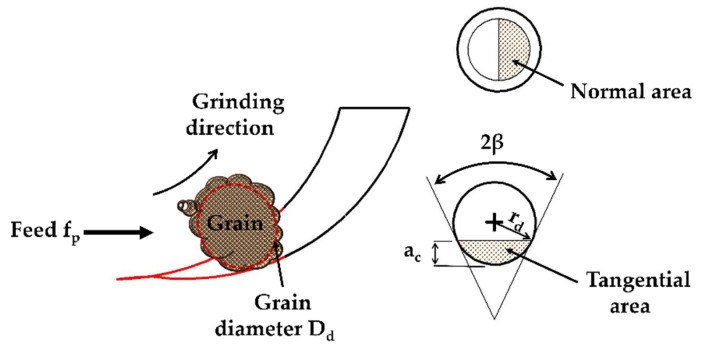
Elastic and plastic projected areas of a single active grain.

As shown in Figure 4, the NT areas depend on the half-vertex angle β of a single grit, the grain diameter D_d_ and cutting depth a_c_ or h_t_. Equation (11) represents the projection area in normal plane A_n-elas_, the area in the thrust axis A_t-elas_ is displayed in Equation (12).

(11) $$A_{\text{n-elas}}=r_{d}{}^{2}\left(\beta -0.5\;sin\;\beta \right)$$

where h_t_ is the cutting depth in the elastic zone and established as $\left(h_{t}=\frac{a_{\max}}{{2N}_{\text{d-x}}}\right)$ (N_d-x_ is the active grains through *x*-axis which is discussed in Section 3.1).

(12) $$A_{\text{t-elas}}=\left(r_{d}{}^{2}\cdot \beta \right)-\left(\left(r_{d}-h_{t}\right)\cdot r_{d}\cdot \sin\beta \right).$$

The angle β is set as a dynamic value calculated based on the grinding modes, it, therefore, depends entirely on the cutting depth. In the elastic mode, the angle β is set as $\left(\beta =\cos^{-1}\left(\frac{D_{d}-{2h}_{t}}{D_{d}}\right)\right)$.

In the plastic region, the projected areas whether in the normal axis A_n-plas_ or the tangential axis A_t-plas_ are established as:

(13) $$A_{\text{n-plas}}=\frac{\pi }{2}\left({2r}_{d}\cdot a_{c}-a_{c}{}^{2}\right)$$

(14) $$A_{\text{t-plas}}=r_{d}{}^{2}\cdot \beta -\left(r_{d}-a_{c}\right)\cdot \sqrt{{2r}_{d}\cdot a_{c}-a_{c}{}^{2}}$$

where the angle β under plastic mode is calculated as $\left(\beta =\cos^{-1}\left(\frac{D_{d}-{2a}_{c}}{D_{d}}\right)\right)$.

## 3. Grinding Force Establishment

Based on the preceding analyses of the material removal, the force model is classified according to the classification of the grinding modes which stated in Equation (3). The sophisticated force pattern when surface grinding can be determined in two parts, namely, NT grinding forces. The grinding force model is constructed according to the individual grain interactivity, thereupon the formula of the active grains should be established initially. Section 3.1 briefly explains how to identify the number of active diamonds.

### 3.1. Active Abrasive Grain

**Figure 5:**
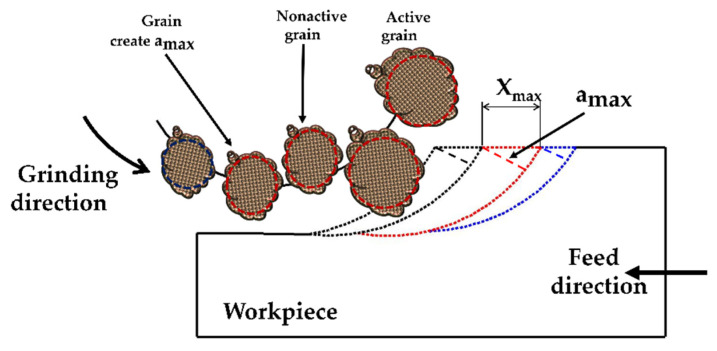
The trajectory and allocation of abrasive grains.

The maximum chip thickness a_max_ is a dynamic concept in the grinding process and every single active grit creates a different value based on the wheel properties and grinding parameters as displayed in Figure 5. For this, not all the grains making protrusion in the surface of the grinding tool will engage in the grinding process. So, the number of active diamonds is calculated separately in the (*x*–*z*) directions, then the total active grits are obtained by multiplying the active grains on *x*-axis and *z*-axis. The number of active diamonds formula is established through identifying the touch arc length λ_c_ as expressed in Equation (15), and the average distance between two active grains λ_r_ is displayed in Equation (9). The touch arc length λ_c_ is defined as [34,35]:

(15) $$\lambda_{c}=\left(1+\frac{f_{p}}{V_{r}}\right)\cdot \sqrt{{2\;h\cdot r}_{t}}.$$

The active grains in the *x*, *z*-axes are explained in the following equations:

(16) $$N_{\text{d-x}}=\frac{\lambda_{c}}{{\;2r}_{d}+\lambda_{r}}=\left(1+\frac{f_{p}}{{\omega \cdot r}_{t}}\right)\cdot \sqrt{\frac{{2C}_{G}\cdot h\cdot r_{t}}{{\pi r}_{d}{}^{2}}}$$

(17) $$N_{\text{d-z}}=\frac{a_{w}}{{2r}_{d}+\lambda_{r}}=\frac{a_{w}}{r_{d}}\cdot \sqrt{\frac{C_{G}}{\pi }}.$$

The total active grains N_d-Tot_ is calculated through multiplying the active grains in *x*-axis N_d-x_ and the active grains in *z*-axis N_d-z_ as:

(18) $$N_{\text{d-Tot}}=N_{\text{d-x}}\cdot N_{\text{d-z}}=\left(1+\frac{f_{p}}{V_{r}}\right)\cdot \left(\frac{C_{G}\cdot a_{w}}{{\pi r}_{d}{}^{2}}\right)\cdot \sqrt{{2\;h\cdot r}_{t}}.$$

### 3.2. Force Model in Ductile Region

**Figure 6:**
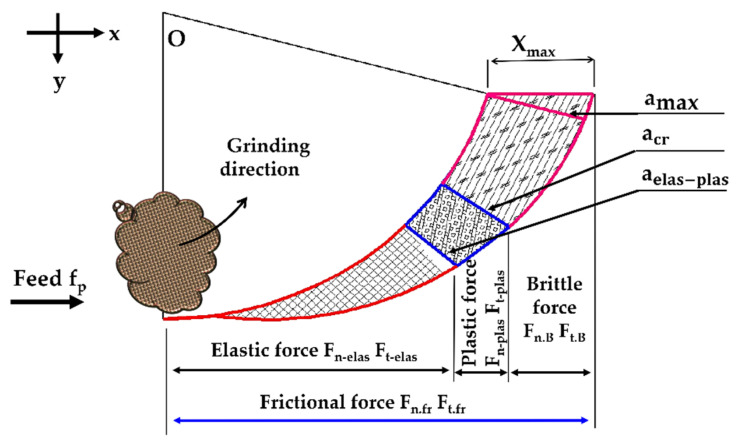
Schematic diagram of grinding force variation during grinding.

As shown in Figure 6, the mode of a ductile zone is classified into two categories; elastic and plastic modes. So, the force model taking the frictional force into a consideration is built according to these modes.

(19) $$\{\begin{array}{c} F_{n}=F_{\text{n-elas}}+F_{\text{n-plas}}+F_{n.\mathrm{fr}}\\ F_{t}=F_{\text{t-elas}}+F_{\text{t-plas}}+F_{t.\mathrm{fr}} \end{array}$$

where F_n_ and F_t_ represent the total grinding force of NT coordinates respectively, F_n-elas_ and F_t-elas_ are the NT rubbing forces acted in the elastic region, F_n-elas_ and F_t-elas_ are the NT plowing forces acted in the plastic region, F_n.fr_ and F_t.fr_ are the NT frictional forces which acted through the whole grinding process.

#### 3.2.1. Elastic Force

When a_c_ < a_elas-plas_ the elastic stage is dominant, the grinding force is named according to the stage name and expressed as follows:

(20) $$F_{\text{n-elas}}=N_{\text{d-Tot}}\cdot P_{\text{max-elas}}\cdot A_{\text{n-elas}}$$

(21) $$F_{\text{t-elas}}=\mu N_{\text{d-Tot}}\cdot P_{\text{max-elas}}\cdot A_{\text{t-elas}}$$

where P_max-elas_ is the maximum contact pressure occurred in the elastic stage, μ is the coefficient of friction.

To calculate the maximum pressure in elastic and plastic zones, the maximum pressure acted in the ductile mode P_max_ must be clarified initially, therefore it is displayed by Equation (22) [36].

(22) $$P_{\max}=\left(\eta +\zeta \left(\frac{{\omega \cdot r}_{t}}{R_{c}\cdot \varepsilon_{c}}\right)\right)\cdot P_{i}$$

where η, ζ are constants defined according to the material sensitivity to strain average. ε_c_ is strain average constant calculated as ε_c_ = 1 [37], P_i_ is the contact pressure related to the pressure in elastic zone P_i-elas_ classified by Equation (24), as well as pressure in the plastic zone P_i-plas_ expressed by Equation (31), R_c_ is the contact arc length identified by Equation (23).

(23) Rc=2(ac·Dd−ac2)12

where D_d_ is the average median diameter of a single abrasive grit.

The pressure in the elastic mode can be classified as [24,38]:

(24) $$P_{\text{i-elas}}=\frac{4\sqrt{2}E_{\mathrm{ef}}}{3\pi }\cdot \sqrt{\frac{a_{c}}{D_{d}}}$$

where the effective elastic modulus E_ef_ can be calculated through Equation (25).

(25) $$E_{\mathrm{ef}}={\left(\frac{1-{\upsilon_{p}}^{2}}{E_{p}}+\frac{1-{\upsilon_{d}}^{2}}{E_{d}}\right)}^{-1}$$

where υ_p_, υ_d_ are the Poisson’s ratio of the workpiece and diamond grain respectively, E_p_, E_d_ are the elastic modulus of the workpiece and diamond grain respectively.

Then the maximum pressure in elastic mode P_max-elas_ can be obtained as follows:

(26) $$P_{\text{max-elas}}=\frac{4\sqrt{2}E_{\mathrm{ef}}}{3\pi }\cdot \left(\eta +\zeta \left(\frac{{\omega \cdot r}_{t}}{R_{c}\cdot \varepsilon_{c}}\right)\right)\cdot \sqrt{\frac{a_{c}}{D_{d}}}.$$

The NT forces that occurred in elastic mode F_n-elas_ and F_t-elas_ are respectively expressed by Equations (27) and (28).

(27) $$F_{\text{n-elas}}=N_{\text{d-Tot}}\cdot \frac{2\sqrt{2}E_{\mathrm{ef}}}{3}\cdot \left(\eta +\zeta \left(\frac{{\omega \cdot r}_{t}}{R_{c}\cdot \varepsilon_{c}}\right)\right)\cdot \sqrt{\frac{a_{c}}{D_{d}}}\cdot \left({2r}_{d}\cdot h_{t}-h_{t}{}^{2}\right)$$

(28) $$F_{\text{t-elas}}=\mu N_{\text{d-Tot}}\cdot \frac{4\sqrt{2}E_{\mathrm{ef}}}{3\pi }\cdot \left(\eta +\zeta \left(\frac{{\omega \cdot r}_{t}}{R_{c}\cdot \varepsilon_{c}}\right)\right)\cdot \sqrt{\frac{a_{c}}{D_{d}}}\cdot \left(\left(r_{d}{}^{2}\cdot \beta \right)-\left(\left(r_{d}-h_{t}\right)\cdot r_{d}\cdot sin\beta \right)\right).$$

#### 3.2.2. Plastic Force

The plastic zone is dominant whenever the a_elas-plas_ < a_c_ < a_cr_. The plowing force is created whenever the chip thickness is analogous to the rim of the active grits radius [39]. Zhang et al. [24] recommended that the ploughing force is fundamentally created due to the plastic deformation in the grinding zone of the workpiece. Accordingly, the contact force due to contact pressure represents the plowing force as long as the plastic mode is dominant.

(29) $$F_{\text{n-plas}}=N_{\text{d-Tot}}\cdot P_{\text{max-plas}}\cdot A_{\text{n-plas}}$$

(30) $$F_{\text{t-plas}}={\mu N}_{\text{d-Tot}}\cdot P_{\text{max-plas}}\cdot A_{\text{t-plas}}.$$

The contact pressure in the plastic mode P_i-plas_ is defined in Equation (31) [40].

(31) $$P_{\text{i-plas}}=€{\left(\frac{H_{p}{}^{4}}{E_{p}}\right)}^{1/3}$$

where € is a unitless factor assumed as € = 1.5 for HBM [40].

Then, the maximum pressure in the elastic mode P_max-plas_ is calculated as:

(32) $$P_{\text{max-plas}}=€\left(\eta +\zeta \left(\frac{{\omega \cdot r}_{t}}{R_{c}\cdot \varepsilon_{c}}\right)\right)\cdot {\left(\frac{H_{p}{}^{4}}{E_{p}}\right)}^{1/3}.$$

Consequently, the NT forces created in the plastic zone are identified through Equations (33) and (34) respectively.

(33) $$F_{\text{n-plas}}=\frac{\pi €}{2}N_{\text{d-Tot}}\left(\eta +\zeta \left(\frac{{\omega \cdot r}_{t}}{R_{c}\cdot \varepsilon_{c}}\right)\right)\cdot {\left(\frac{H_{p}{}^{4}}{E_{p}}\right)}^{1/3}\cdot \left({2r}_{d}\cdot a_{c}-a_{c}{}^{2}\right)$$

(34) $$F_{\text{t-plas}}=\mu €N_{\text{d-Tot}}\left(\eta +\zeta \left(\frac{{\omega \cdot r}_{t}}{R_{c}\cdot \varepsilon_{c}}\right)\right)\cdot {\left(\frac{H_{p}{}^{4}}{E_{p}}\right)}^{1/3}\cdot \left(r_{d}{}^{2}\cdot \beta -\left(r_{d}-a_{c}\right)\cdot \sqrt{{2r}_{d}\cdot a_{c}-a_{c}{}^{2}}\right).$$

### 3.3. Force Model in the Brittle Region

(35) $$\{\begin{array}{c} F_{n}=F_{\text{n-elas}}+F_{\text{n-plas}}+F_{n.\mathrm{fr}}+F_{n.B}\\ F_{t}=F_{\text{t-elas}}+F_{\text{t-plas}}+F_{t.\mathrm{fr}}+F_{t.B} \end{array}$$

where F_n.B_ and F_t.B_ are the NT grinding forces that occurred in the brittle tear region defined through Equations (36) and (37) respectively.

(36) $$F_{n.B}=N_{\text{d-Tot}}\cdot N\cdot T_{\mathrm{ef}}\cdot F_{B.\max}$$

(37) $$F_{t.B}=\mu F_{n.B}$$

where N is the rotational speed of the spindle, T_ef_ is the effective grinding time of single grinding grain for one cycle expressed in Equation (38), F_B.max_ is the maximum force in the brittle region.

(38) $$T_{\mathrm{ef}}=\frac{L_{\mathrm{ef}}}{V_{r}}$$

that L_ef_ is the effective length between the grinding rod and grinding surface expressed as:

(39) $$L_{\mathrm{ef}}=\frac{L_{D}\cdot L_{s}}{L_{D}+L_{s}}$$

where L_S_ and L_D_ are the geometric and dynamic contact lengths between the grinding tool and workpiece grinding surface as shown in Figure 2 and modeled by Equations (40) and (41) respectively.

(40) $$L_{S}=r_{t}\cos^{-1}\left(\frac{r_{t}-h}{r_{h}}\right)$$

(41) $$L_{D}=\int_{0}^{T_{\mathrm{Tot}}}\sqrt{V_{P}{}^{2}+{\left({2\;r}_{t}\;\omega \;V_{P}\;\cos\;\left(\mathrm{wt}\right)\right)}^{2}+{\left(r_{t}\;\omega \right)}^{2}}\mathrm{dt}$$

where T_Tot_ is the total grinding time for one active grit to intersect the contact length L_D_ calculated as:

(42) $$T_{\mathrm{Tot}}=\frac{\;\cos^{-1}\left(\frac{r_{t}-h}{r_{t}}\right)}{2\pi N}.$$

The maximum force created in the brittle zone is classified by Equation (43) [41].

(43) $$F_{B.\max}=\sqrt{\frac{{8D}_{d}\cdot a_{\max}{}^{3}}{9}\cdot {\left(\frac{E_{p}}{1-{\upsilon_{p}}^{2}}\right)}^{2}\;}=\frac{{2E}_{p}}{3(1-{\upsilon_{p}}^{2})}\cdot \sqrt{{2D}_{d}\cdot a_{\max}{}^{3}}.$$

By substituting Equations (38) and (43) into Equations (36) and (37), F_n.B_ can be obtained as:

(44) $$F_{n.B}=N_{\text{d-Tot}}\cdot \frac{{2E}_{p}\cdot {N\cdot L}_{D}\cdot L_{s}}{{3V}_{r}(1-{\upsilon_{p}}^{2})\cdot (L_{D}+L_{s})}\cdot \sqrt{{2D}_{d}\cdot a_{\max}{}^{3}}.$$

### 3.4. Frictional Force

In surface grinding of the HBM, the frictional effects are created due to two resources between the abrasives and the grinding surface, one of them is produced by the flux of workpiece over the touch face where the other is generated from the frictional between the wear protrusion of grits and the grinding face. The influence of grinding heat affects the coefficient of friction to vary with varying conditions and behavior of surface grinding. Therefore, modeling the coefficient of friction considering all of these factors is a highly significant task.

(45) $$F_{n.\mathrm{fr}}=N_{\text{d-Tot}}\cdot P_{\mathrm{cont}}\cdot A_{\mathrm{cont}}$$

(46) $$F_{t.\mathrm{fr}}=\mu F_{n.\mathrm{fr}}$$

where P_cont_ and A_cont_ are the contact pressure and contact area between the wear protrusion of active grits and the grinding face respectively.

Through the convergence of the parabolic function with the cutting path, the perversion is located between the arc of the grinding channel and the radius of the grinding wheel and can be calculated by Equation (47) [42].

(47) $$\rho =\frac{{2f}_{p}}{r_{t}\cdot V_{r}}.$$

In the formula, ρ is the deviation between the arc of the grinding channel and the radius of the grinding rod. 

The average touch pressure between a single grit and workpiece P_cont_ is expressed by Equation (48) [43]:

(48) $$P_{\mathrm{cont}}=\rho P_{0}=\frac{{2P}_{0}\cdot f_{p}}{r_{t}\cdot V_{r}}$$

where P_0_ is defined as the experiential suitability factor calculated via the experiments.

The frictional coefficient μ is clarified as [44].

(49) $$\mu =\frac{\alpha_{1}}{P_{\mathrm{cont}}}+\alpha_{2}.$$

That α_1_, α_2_ represent the coefficients of the materiality of the frictional pairs.

(50) $$A_{\mathrm{cont}}=\int_{0}^{r_{d\;}}\left(2\beta \;r_{d}\right){\mathrm{dr}}_{d}-\left(r_{d}\sin\beta \right)\cdot \left(r_{d}\cos\beta \right)=\frac{1}{8}D_{d}{}^{2}\cdot \left(2\beta -\sin2\beta \right).$$

By substituting Equations (48) and (50) into Equations (45) and (46), F_n.fr_ can be calculated as:

(51) $$F_{n.\mathrm{fr}}=N_{\text{d-Tot}}\cdot \frac{P_{0}\cdot f_{p}\cdot D_{d}{}^{2}}{4r_{t}\cdot V_{r}}\cdot \left(2\beta -\sin2\beta \right).$$

In our study, the coefficient of friction is calculated through Equation (52) [45].

(52) $$\mu =0.3885-0.011\ln({1000\;V}_{r}).$$

## 4. Predicted Force Model Analysis

In the preceding parts, the arithmetic model of surface grinding force for HBM has been suggested, whilst in the current part, the effects of grinding conditions on the grinding force will be studied through the suggested model.

**Figure 7:**
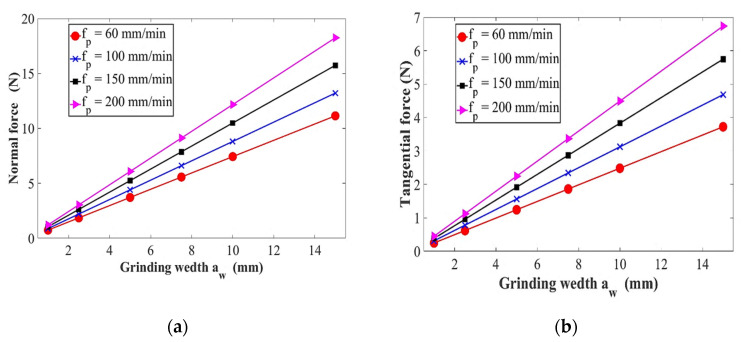

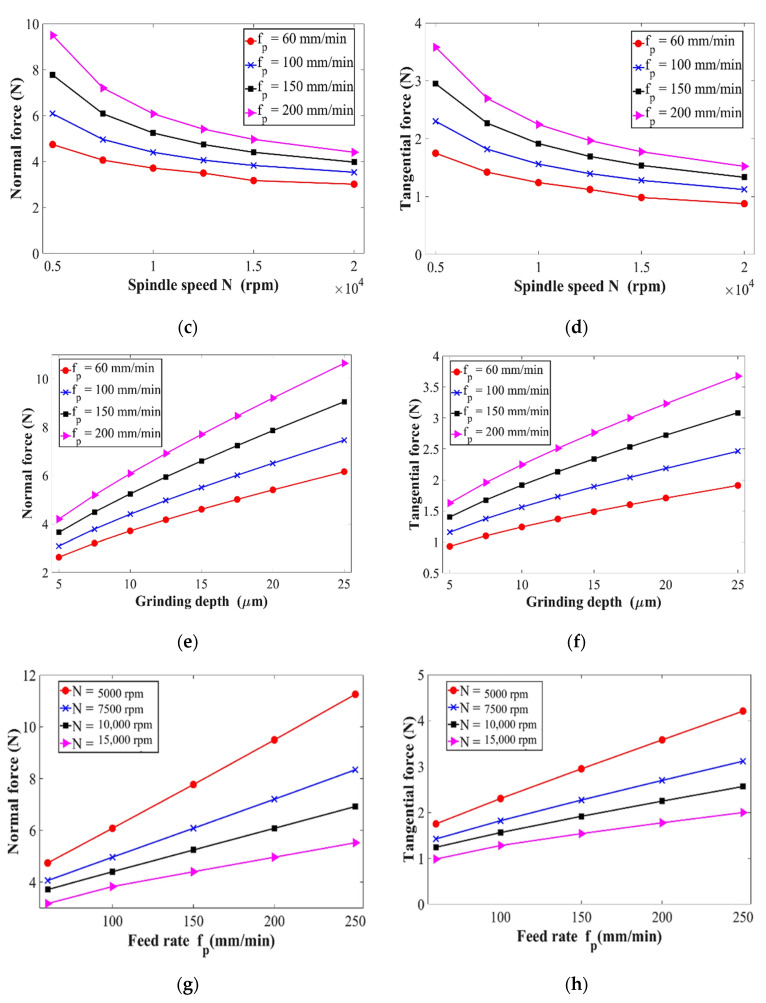
Calculated grinding forces (**a**) N = 10^4^ min^−1^, h = 10 µm, r_t_ = 2 mm; (**b**) N = 10^4^ min^−1^, h = 10 µm, r_t_ = 2 mm; (**c**) h = 10 µm, a_w_ = 5 mm, r_t_ = 2 mm; (**d**) h = 10 µm, a_w_ = 5 mm, r_t_ = 2 mm; (**e**) N = 10^4^ min^−1^, a_w_ = 5 mm, r_t_ = 2 mm; (**f**) N = 10^4^ min^−1^, a_w_ = 5 mm, r_t_ = 2 mm; (**g**) h = 10 µm, a_w_ = 5 mm, r_t_ = 2 mm; (**h**) h = 10 µm, a_w_ = 5 mm, r_t_ = 2 mm.

Surface grinding parameters are listed in Table 1 for grinding force predictions. The relationship between grinding conditions (f_p_, a_w_, N, h) and NT grinding forces are displayed in Figure 7 according to the derived equations. The average amplitude of the NT forces is almost the same. By observing the force signal curves of the grinding process, it is found that both are the same. This is mainly because of feed speed is very low compared to the rotational speed of the abrasive particles, therefore it has little effect on the grinding force. As shown in Figure 7, the curves of the NT forces are varying with machining parameters during the grinding process. When the abrasive grain maintains its original shape, as the grinding depth h increases, the grinding force increases monotonically, and the NT forces change roughly the same; on the other hand, as the grinding speed increases, the grinding force gradually and nonlinearly decreases. This is consistent with the conclusion that the grinding speed is increased and the cutting force of a single abrasive particle is reduced in the current research on high-speed grinding processing [46]. In short, both NT grinding forces increase with the ascending of grinding conditions of feed rate f_p_, grinding depth h, and grinding width a_w_; however, decreasing with increasing of spindle speed.

## 5. Experimental Validation

### 5.1. Experimental Setup

**Figure 8:**
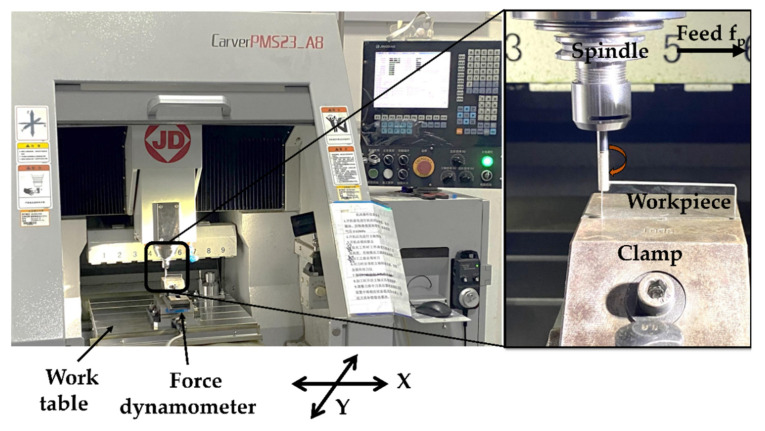
The precision surface grinder CarverPMS23_A8.

To validate the arithmetical force model, the experiments were carried out on the precision surface grinder CarverPMS23_A8 (Figure 8). In the experiments, the substrate rotates at a certain speed to realize the grinding of a single abrasive grit where the maximum experimental speed of the spindle was 1.5 × 10^4^ min^−1^, and the spindle drives the grinding wheel to move longitudinally to adjust the feed rate. The NT forces data is obtained using a high resolution, high precision dynamometer.

The workpiece material used is K9 glass, with the dimensions of 50 mm × 20 mm × 3 mm. Table 2 lists the K9 glass ingredients where its material properties are listed in Table 3. The 700 # glass bonded grinding top is selected in the experiments with a diameter of 4 mm, while the abrasive grits are distributed over the 15 mm of tool length, grinding wheel specifications are listed in Table 4.

The diameter size of the grinding rod is affected by the grinding parameters especially when the rotational spindle speed more than 50 × 10^4^ min^−1^, the diameter is equal or less than 1 mm, and the mesh size is almost less than 400#, all these conditions have adverse effects on the accuracy of grinding [33]. To avoid any possible deviation in the chip thickness calculations due to a decrease in the diameter of the grinding wheel, all these conditions will be taken into consideration in the current study.

### 5.2. Experimental Force

In the experimental part, Table 5 presents the grinding conditions. Kistler 9257 B dynamometer is used to gauge the grinding forces.

**Figure 9:**
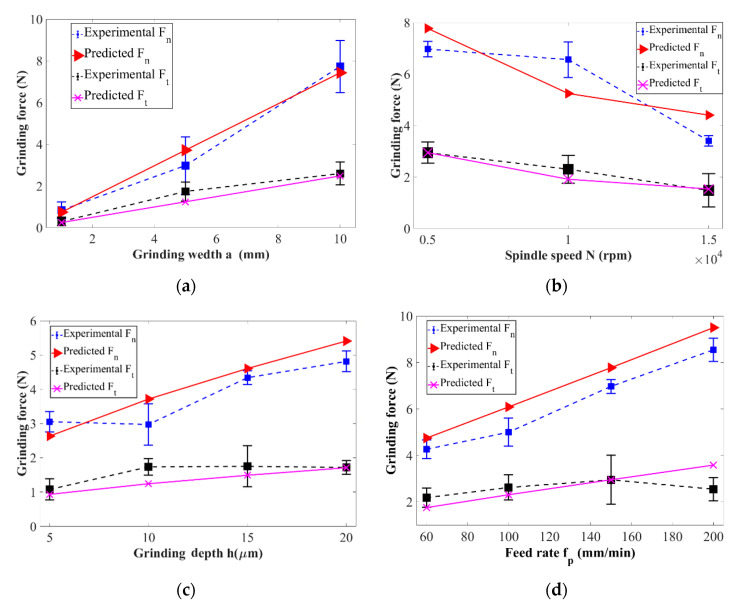
Experimental grinding forces at: (**a**) N = 10,000 min^−1^, h = 10 µm, r_t_ = 2 mm, f_p_ = 60 mm/min; (**b**) a_w_ = 5 mm, h = 10 µm, r_t_ = 2 mm, f_p_ = 150 mm/min; (**c**) h = 10 µm, a_w_ = 5 mm, r_t_ = 2 mm, f_p_ = 60 mm/min; (**d**) N = 5000 min^−1^, h = 10 µm, a_w_ = 5 mm, r_t_ = 2 mm.

The arithmetical model of the grinding force is established by assuming that the active grains on the grinding wheel surface are randomly distributed and the shape of all the grains is consistent. Besides, the workpiece materials selected in this study are considered as HBM, and the material properties will not be affected by the grinding conditions. The curves below presented in Figure 9 are the comparison between arithmetic values and measurement values of NT grinding forces under traditional surface-grinding conditions. It can be seen that the theoretical calculation results are consistent with the experimental outputs. The grinding target is to achieve high-quality surface and subsurface integrity, therefore the grinding force and grinding parameters must be under full control to achieve this goal. The precision of the prediction force represented in the mathematical model, grinding wheel conditions of the surface grinding process, fineness location of the workpiece, the ability to purify the measured grinding force through the output results are the essential reasons which create the deviations between the predicted force model and the experimental results.

### 5.3. Workpiece Topography

**Figure 10:**
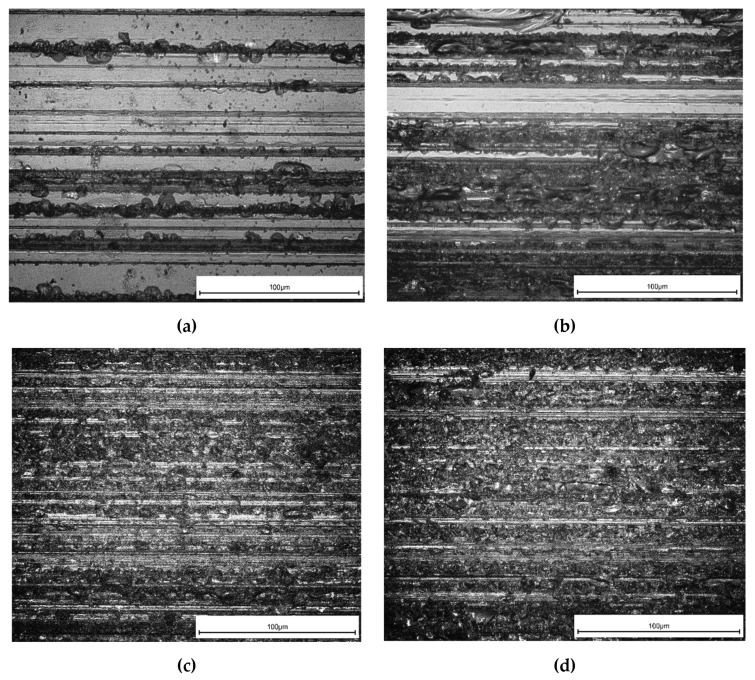
K9 surface topography at: (**a**) h = 1 µm, N = 10^4^ min^−1^, f_p_ = 60 mm/min; (**b**) h = 5 µm, N = 10^4^ min^−1^, f_p_ = 100 mm/min; (**c**) h = 10 µm, N = 10^4^ min^−1^, f_p_ = 60 mm/min; (**d**) h = 15 µm, N = 1.5 × 10^4^ min^−1^, f_p_ = 100 mm/min.

One of the most paramount demands in the industrial implementation is the quality of face topography, which can be counted as a leading indicator of production fineness. Figure 10 displays the workpiece surface captures taken by the ZEISS AXIO microscope apparatus. The captures are taken based on the grinding depth, the surface displayed in Figure 10a, which has a less grinding depth, seems to be better than Figure 10b–d. In conclusion, it can be clarified that brittle tear is the primary mode of elimination gradually with increasing chip thickness. Subsequently, the classifications of the material removal regions discussed in Section 2.2 are more agreeable.

## 6. Conclusions

K9 glass has a high hardness and low fracture toughness which greatly fluctuates the cutting force generated during the machining process. Therefore, achieving the efficient prediction and controlling of the grinding force during the processing of optical glass is of great importance to improve the processing efficiency and the quality of the processed surfaces. The investigation model of the grinding force consists of ductile, ductile-to-brittle, brittle forces. The suggested force model is taking the two sources of friction into a consideration, as well as considers the elastic–plastic transition, randomly distributed of active grain, and the tool diameter variation during processing. Moreover, the predicted model is evaluated by comparing the experimental measurement results to the model prediction values. The main conclusions and results of this paper are as follows:

- The force model was verified by the K9 glass grinding test with a fixed abrasive grain through different grinding parameters. The theoretical analysis results have the same trend as the experimentally measured values, which proves that the model is reliable.
- Based on the analysis of the protruding height and horizontal distribution characteristics of abrasive grits on the grinding tool surface, the a_max_ is a dynamic concept, which has different values during grinding.
- Under the same grinding conditions, the grinding force inclines with the rotational speed N growing, where it decreases by growing the grinding depth h, grinding width a_w_, and feed rate f_p_. Besides, the conjunction impacts between the number of active grits and grit size led to the grinding force showing an irregular direction in the force model.
- The normal force is more than the tangential force during surface grinding of HBM, for that reason, controlling the forces in the normal axis is more significant.

Eventually, the current model is capable of fully representing the actual material removal attitudes and effectively predicting the NT forces when grinding the HBM surfaces as well.

## Figures and Tables

**Table 1:** Surface grinding conditions for predicted grinding force.

Group No	Feed Rate f_p_ (mm/min)	Spindle Speed N (min^−1^) × 10^3^	Grinding Depth h (µm)	Grinding Width a_w_ (mm)	Wheel Diameter D_t_ (mm)
1	60, 100, 150, 200	10	10	1, 2.5, 5, 7.5 10, 15	4
2	60, 100, 150, 200	5, 7.5, 10, 12.5, 15, 20	10	5	4
3	60, 100, 150, 200	10	5, 7.5, 10, 12.5, 15, 17.5, 20	5	4
4	60, 100, 150, 200, 250	5, 7.5, 10, 12.5, 15, 20	10	5	4

**Table 2:** Chemical compositions of K9 optical glass.

Element	SiO_2_	B_2_O_3_	BaO	Na_2_O	K_2_O	As_2_O_3_
Content (wt%)	69.13	10.75	3.07	10.40	6.29	0.36

**Table 3:** Material properties of K9 glass used in the experiments.

Material	Dispersion	Elastic Modulus (GPa)	Fracture Toughness (MPa·m^1/2^)	Vickers Hardness (GPa)	Poisson Ratio
K9	0.00806	88.5	2.63	7.8	0.203

**Table 4:** Grinding wheel specifications used in the experiments.

Type	Mesh	Wheel Diameter D_t_	Grain Diameter D_d_	Elastic Modulus	Poisson Ratio
PCD	700#	4 mm	21.8 µm	800 GP	0.07

**Table 5:** Experimental conditions.

Group No	Feed Rate f_p_ (mm/min)	Spindle Speed N (min^−1^) × 10^3^	Grinding Depth h (µm)	Grinding Width a_w_ (mm)	Tool Diameter D_t_ (mm)
1	60	10	10	1, 5, 10	4
2	150	5, 10, 15	10	5	4
3	60	10	5, 10, 15, 20	5	4
4	60, 100, 150, 200	5	10	5	4

## Nomenclature

€	Dimensionless constant related to HBM
µ	Coefficient of friction
a_c_	Undeformed chip thickness
a_elas-plas_, a_cr_	Chip thickness of elastic-to-plastic, and ductile-to-brittle transitions
a_max_	Maximum chip thickness
A_n-elas_, A_t-elas_	Normal and tangential areas in elastic zone respectively
A_n-plas_, A_t-plas_	Normal and tangential areas in plastic zone respectively
a_w_	Grinding width
C_G_	Size fracture of grinding grit.
E_p_, E_d_, E_ef_	Workpiece, abrasive grain, and effective modulus of elasticity respectively
F_B.max_	Maximum brittle force
F_n_, F_t_	Total normal and tangential grinding force
F_n.B_, F_t.B_	Normal and tangential brittle force
F_n.fr_, F_t.fr_	Normal and tangential frictional force
F_n-elas_, F_t-elas_	Normal and tangential force occurred on elastic zone
F_n-plas_, F_t-plas_	Normal and tangential force occurred on plastic zone
f_p_	Feed rate
H_p_	Workpiece hardness
h_t_	Chip thickness in the elastic zone
K_IC_	Fracture toughness of workpiece material
L_S_, L_D_, L_ef_	Geometric, dynamic, and effective contact length between worksurface and grinding tool
N	Spindle speed
N_d-x_, N_d-y_, N_d-Tot_	*x*-axis, *y-*axis, and total active grains share in grinding
P_0_	Experiential suitability factor
P_cont_, A_cont_	Contact pressure and the contact area between the wear protrusion of grits and the grinding face
P_max_	Maximum contact pressure
P_max-elas_, P_max-plas_	Maximum pressure created on worksurface through plastic and elastic regions respectively
R_c_	Arc contact length between single grain and worksurface
r_d_, D_d_	Mean radius and diameter of grinding grain
r_t_	Grinding wheel radius
S(t)	Displacement of a single grit
t	Grinding time
T_ef_	Effective time
T_Tot_	The total grinding time of grinding tool to intersect the dynamic touch length in one cycle
V_r_	The relative velocity of a single grain relative to the workpiece surface
X_max_	Maximum axial displacement of single abrasive grit in one cycle
α_1_, α_2_	Coefficients of the materiality of the frictional pairs
β	The half-oblique angle of a single active grit
ε, ε_c_	Unitless constants
η, ζ	Constants defined according to the material sensitivity to strain average
λ_c_	Touch arc length
λ_r_	Average space between any two active grains
ρ	Deviation of the arc of the grinding channel and the radius of the grinding rod
υ_p_, υ_d_	Poisson’s ratios of workpiece and grit respectively
ω	Angular velocity
